# Cardiac mechanotransduction from development to disease

**DOI:** 10.1063/5.0320788

**Published:** 2026-04-01

**Authors:** Na Yeon Kim, Hyojung Jo, Chloe Becker, Isaiah Soicher, Yeqing Ni, Sangkyun Cho, Hyeonyu Kim

**Affiliations:** 1 The Department of Bioengineering, Northeastern University, Massachusetts 02115, USA; 2 The Department of Mechanical and Industrial Engineering, Northeastern University, Massachusetts 02115, USA; 3 The Department of Biochemistry, Northeastern University, Massachusetts 02115, USA; 4 The Department of Chemical and Biomolecular Engineering, Johns Hopkins University, Maryland 21218, USA; 5 Institute for NanoBiotechnology, Johns Hopkins University, Maryland 21218, USA; 6 Institute for Mechanobiology, Northeastern University, Massachusetts 02115, USA

## Abstract

Mechanical cues control key aspects of cardiac structure formation and function from heart development through adult life. Because the heart is a pump, forces from muscle contraction and blood flow generate normal and shear stresses that, together with matrix stiffness, regulate cell fate, growth, and homeostasis through mechanotransduction. This review describes how mechanosensors in cardiomyocytes, endothelial cells, and fibroblasts, including integrins and stretch-activated ion channels, couple mechanical stimuli to their cellular responses. We outline pathways that translate force into key phenotypes relevant to morphogenesis, homeostasis, and disease progression, with emphasis on RhoA/ROCK, calcium, and Yes-associated protein (YAP) signaling. We also explain how elevated mechanical load driven by hypertension activates hypertrophic and fibrotic remodeling of cardiac chambers, particularly through transforming growth factor-β, integrins, YAP, and calcineurin signaling. Finally, we highlight emerging roles for mechanosensitive microRNAs in coordinating proliferation, metabolism, electrophysiology, and extracellular matrix dynamics in the heart. Since most, if not all, of these pathways are interconnected, a comprehensive understanding will require high-resolution maps of cardiac mechanical environments and clear links between defined stimuli and cell-type-specific responses. These insights will advance fundamental understanding and guide the development of more effective therapeutic strategies.

NOMENCLATURE

ACE: Angiotensin-converting enzyme
AMPK: AMP-activated protein kinase
AT1R: Angiotensin II type 1 receptor
AJ: Adherens junction
Arp2/3: Actin-related protein 2/3 complex
AVC: Atrioventricular canal
BNP: Brain natriuretic peptide
CaMKII: Calcium/calmodulin-dependent kinase II
CFD: Computational fluid dynamics
DCM: Dilated cardiomyopathy
DGC: Dystrophin glycoprotein complex
DMD: Duchenne muscular dystrophy
ECM: Extracellular matrix
EndoMT: Endothelial-to-mesenchymal transition
eNOS: Endothelial nitric oxide synthase
ErbB2/ErbB4 Erb-B2: Receptor tyrosine kinase 2 and 4
ERK1/2: Extracellular signal-regulated kinase 1/2
FAK: Focal adhesion kinase
FEM: Finite element model
GJ: Gap junction
HCM: Hypertrophic cardiomyopathy
HLHS: Hypoplastic left heart syndrome
ICD: Intercalated disk
IL-6: Interleukin-6
iPSC: Induced pluripotent stem cell
JNK: c-Jun N-terminal kinase
KLF: Krüppel-like factor
LIMK1/2: LIM domain kinase 1 and 2
LINC: Linker of nucleoskeleton and cytoskeleton
MAPK: Mitogen-activated protein kinase
MCP: Monocyte chemoattractant protein
MRI: Magnetic resonance imaging
MRTF-A: Myocardin-related transcription factor A
NFAT: Nuclear factor of activated T cells
NLRP3: NOD-like receptor family pyrin domain-containing 3
NRVM: Neonatal rat ventricular cardiomyocyte
N-WASP: Neural Wiskott–Aldrich syndrome protein
OFT: Outflow tract
PECAM1: Platelet endothelial cell adhesion molecule 1
PIP2: Phosphatidylinositol 4,5-bisphosphate
PIP5K: Phosphatidylinositol 4-phosphate 5-kinase
RAAS: Renin-angiotensin-aldosterone system
RhoA: Ras homolog family member A
ROCK: Rho-associated kinase
ROS: Reactive oxygen species
RyR: Ryanodine receptors
SAC: Stretch-activated ion channel
SGK1: Serum/glucocorticoid-regulated kinase 1
SRC: Proto-oncogene tyrosine-protein kinase Src
TAC: Transverse aortic constriction
TAZ: Transcriptional coactivator with PDZ-binding motif
TGF-β: Transforming growth factor-β
TEAD: Transcriptional enhanced associate domain
TRP: Transient receptor potential
TRPP2: Transient receptor potential polycystin 2
TRPV4: Transient receptor potential vanilloid 4
VE-cadherin: Vascular endothelial cadherin
VEGF: Vascular endothelial growth factor
YAP: Yes-associated protein

## I. INTRODUCTION

The heart is the body's most active organ, moving unceasingly throughout life. This motion is driven by intrinsic forces generated by cardiomyocytes, producing blood flow throughout the body and within the cardiac chambers. From embryonic development through adulthood, these forces create an essential mechanical milieu for cardiac cells. Together with biochemical cues, physical stimuli regulate cardiac development, maintain homeostasis, and contribute to disease through mechanotransduction.^1^ A more complete understanding of mechanotransduction requires attention beyond the organ-level, to age, cell type, and anatomic region within the heart, because mechanical environments differ across these factors and so do the governing signaling pathways.

In this review, we provide a comprehensive overview of mechanical cues in the heart under diverse conditions and the associated mechanical signaling. We first highlight dynamic forces during morphogenesis across developmental stages, including the formation of chambers and valves. We then describe the physical environment of the healthy adult heart at the surfaces and within the ventricular wall. Next, we examine how abnormal states such as hypertension alter these environments and activate pathological mechanical signaling, focusing on hypertrophy and fibrosis. In cardiac fibrosis, stiffening of the extracellular matrix (ECM) introduces additional abnormal cues that further modify cell behavior. Across contexts, we discuss key mechanosensors, including integrins and stretch-activated ion channels (SACs), that couple mechanical inputs to cellular function. Finally, we consider emerging evidence that small non-coding RNAs, particularly microRNAs (miRNAs), participate in these control processes, which is critical for a complete understanding of cardiac mechanobiology.

## II. CARDIAC DEVELOPMENT AND MORPHOGENESIS

### A. Early flow sensing and endocardial mechanotransduction

During fetal heart development, diverse and dynamic mechanical stresses serve as important regulators of morphogenesis. As the embryonic heart begins to contract and initiate blood flow, cardiomyocytes and endothelial cells are subjected to cyclic mechanical stresses that influence cell-ECM interactions, cytoskeletal reorganization, and cell morphology.^2–6^ In particular, fluid shear stress generated by blood flow is sensed by endothelial cells, including endocardial cells, via SACs such as Piezo1 and the transient receptor potential (TRP) family. These channels transduce membrane tension and fluid shear stress into intracellular calcium signals, triggering downstream signaling cascades.^7–10^

Piezo1 has been shown to increase intracellular Ca^2+^ in embryonic endothelial cells, activating calcium/calmodulin-dependent protein kinase II (CaMKII), mitogen-activated protein kinase kinase kinase 3 (MEKK3), and extracellular signal-regulated kinase 5 (ERK5). This activation upregulates shear-induced expression of Krüppel-like factor (KLF) 2/4.^7^ In addition, genetic ablation of KLF2 results in embryonic lethality with atrioventricular cushion defects, impaired endocardial-to-mesenchymal transition (EndoMT), myocardial thinning, and high-output cardiac failure. Endocardial KLF2 deficiency disrupts valve maturation through reduced Wnt9b expression and canonical Wnt signaling. KLF4 deletion likewise leads to reduced cardiac output, growth retardation, and postnatal lethality.^11–14^

Cardiac mechanosensors for fluid shear stress activate downstream pathways, including Wnt, Notch, and Yes-associated protein (YAP) signaling.^15,16^ These signaling cascades converge on the deposition and swelling of the cardiac jelly, a hydrated ECM layer between the endocardium and myocardium enriched in collagens, proteoglycans, and glycosaminoglycans. This provides the swelling pressure required for endocardial cushion expansion, trabeculation, and valve morphogenesis. In zebrafish and mouse embryos, deletion of endothelial Piezo1 abolished flow-induced KLF2 and downstream Notch1 signaling, resulting in failure of cushion expansion. It also induced a marked reduction in cardiac jelly deposition, accompanied by hypoplastic trabeculae and defective atrioventricular valve primordia.^17^ In human embryonic hearts, single-cell RNA sequencing across the transition from linear tube to chambered structures revealed stage-specific activation of YAP/Transcriptional coactivator with PDZ-binding motif (TAZ) target genes, including COL1A1, COL3A1, and VCAN. These changes coincided with periods of ECM accumulation and chamber expansion, implicating YAP/TAZ as key mediators of cardiac jelly formation and thickening.^18^

The TRP family also has important roles in valve formation. TRPV4 translates shear stress into cardiac development through Ca^2+^-dependent mechanisms. Kondapalli *et al.* demonstrated that endothelial TRPV4 amplifies shear stress-induced Ca^2+^ signals through Ca^2+^ release at inositol 1,4,5-trisphosphate (IP3) receptors, which mediates transforming growth factor-β (TGF-β)-induced EndoMT via the Rho/Snail pathway.^19^ TRPP2 is another significant mechanosensor required for proper endocardial function and for valve morphogenesis. A zebrafish study showed that a TRPP2 mutation markedly reduced proliferation of endocardial cells, accompanied by defective or absent valve leaflets.^20^ Studies in mouse endocardial cells also showed that genetic knockdown or pharmacological blockade of TRPV4 and TRPP2 impaired YAP/TAZ activation and suppressed transcription of ECM genes, including collagens and glycosaminoglycan biosynthetic enzymes, resulting in diminished cushion swelling and valve leaflet formation.^17^ YAP/TAZ activity also promotes EndoMT, allowing endocardial cells to invade the cardiac jelly.

The beating embryonic heart produces high shear stress in the narrow atrioventricular canal (AVC) and outflow tract (OFT) regions, and 4D-flow mapping using Doppler optical coherence tomography (Doppler OCT) confirms that sites of elevated shear correspond precisely to future valve locations.^21^ In addition, recent zebrafish and mouse studies have demonstrated that the AVC endocardium is exposed to oscillatory or disturbed shear stress arising from rhythmic flow reversal as the inflow/outflow valves begin to form, whereas the OFT endocardium is subjected to relatively more laminar shear stress. These distinct flow profiles elicit different endocardial responses. Particularly, the high shear stress in the AVC leads to increased membrane lipid microdomains and enhanced membrane rigidity of endocardial cells.^22^ This alteration in membrane lipid microstructure activates mechanosensitive mechanistic target of rapamycin complex 2 (mTORC2)-protein kinase C (PKC)-Notch signaling pathway by stimulating Notch cleavage, thereby enhancing the AVC cushion formation through Notch-dependent EndoMT.^23,24^

In contrast, in the OFT, oscillatory or pulsatile flow components that arise before valve closure can induce EndoMT via Notch1b signaling. For example, elevated wall shear stress in the zebrafish OFT increases Notch1b activity and drives EndoMT until functional valve leaflets emerge.^25,26^ However, regions of the OFT subjected to high, steady laminar shear stress begin to upregulate classic flow-responsive endothelial factors, such as KLF2 and endothelial nitric oxide synthase (eNOS). This mechanotransduced “arterial” gene program stabilizes the endocardium and, in turn, attenuates further EndoMT, thereby facilitating OFT cushion maturation.^27^ Flow-mediated signals, such as vascular endothelial growth factor (VEGF) and Wnt/β-catenin, are also thought to contribute to this arterial differentiation of the endocardium, reinforcing an anti-EndoMT, endothelial phenotype as the OFT remodels into mature outflow vessels.^28,29^ These results highlight the critical role of mechanosensitive control in cardiac valve development.

In addition to valve formation, fluid shear stress is also important for dynamic morphogenetic transitions such as heart looping and ventricular trabeculation. In particular, blood flow both increases nitric oxide (NO) production and suppresses endothelin 1 (ET1) expression during the dramatic shape changes.^30^ The primitive cardiac tube starts as a linear structure but transforms into a C-shape through heart looping.^31^ In embryonic chicken hearts, high laminar shear stress regions, such as the outer curvature of the looping heart tube, show robust expression of KLF2 and eNOS, which promote a quiescent endothelial phenotype. In contrast, areas subjected to low or oscillatory shear stress, like the inner curvature, show preferential upregulation of ET1 and inflammatory pathways.^32,33^

During heart looping, increased fluid shear stress on the early endocardium elevates tension in its intercellular junctions. This hemodynamic stress induces reorganization of junctional proteins such as platelet endothelial cell adhesion molecule 1 (PECAM1) and vascular endothelial (VE)-cadherin and remodeling of the actin cytoskeleton,^34^ as well as redistribution of ZO-1 and N-cadherin in valve precursor endocardial cells.^35^ Not only SACs, but also these intramembrane complexes help endothelial cells sense shear stress.^36–38^ Mechanical forces directly transmitted from PECAM1 activate VEGFR2 through the recruitment of VE-cadherin, which then activates integrin αvβ3 for binding to ECM and facilitates the alignment of endothelial cells in the direction of flow via cytoskeletal remodeling.^39–41^ This ensures that endothelial cells coordinate their growth and orientation as the tube bends and twists during the looping.

During cardiogenesis, ventricular trabeculation is characterized by the formation of muscular ridges that protrude into the lumen of the primitive ventricles. Clusters of myocardial cells delaminate from the myocardium and invade the cardiac jelly toward the lumen, creating a sponge-like network of myocardial fibers.^42^ This trabecular meshwork increases the surface area for oxygen and nutrient exchange in the embryonic heart before the development of the coronary vasculature.^43^ The initiation of trabeculation is tightly regulated by mechanical cues and endocardium-myocardium interaction. Early embryonic heart contractions and blood flow provide mechanical stimuli that activate Notch signaling in endocardial cells through ciliary flow sensation, initiating downstream pathways required for trabecular growth.^44–47^ Notably, neuregulin 1 (NRG1), expressed by Notch-active endocardial cells, binds to Erb-B2 receptor tyrosine kinase 2 and 4 (ErbB2/ErbB4) receptors on adjacent cardiomyocytes and potently stimulates their proliferation and differentiation into trabecular myocardium.^48^

Mechanical forces not only initiate trabeculation but also directly modulate cellular behavior throughout the process. The blood flow generates laminar shear stress along smooth endocardial surfaces, and disturbed recirculating flow with localized vortices once trabecular ridges protrude into the lumen.^6,21,49^ Under unidirectional shear stress, YAP/TAZ undergo transient nuclear shuttling, whereas disturbed flow induces sustained nuclear localization and activation of proliferation and junctional remodeling.^39^

### B. Computational characterization of fetal hemodynamics

Previous studies highlight blood flow in the fetal heart as a critical regulator at multiple stages of cardiogenesis. Consequently, abnormal intracardiac flow during development may increase the risk of congenital structural heart diseases. To more precisely define the role of flow and to enable early detection of abnormal cardiogenesis, accurate characterization of intracardiac hemodynamics during heart development is highly valuable. Recently, fetal four-dimensional flow magnetic resonance imaging (4D-flow MRI) has been introduced to achieve a more detailed understanding of cardiac function in the human fetus.^50^ This technique enables quantitative hemodynamic measurements within major cardiac vessels, particularly the aorta.^51^ However, significant challenges remain, including the small size of the fetal heart and unavoidable fetal motion during image acquisition, which limit accurate image reconstruction and detailed flow analysis. At present, most measurements are restricted to the aorta.^52^ Extending fetal 4D-flow MRI to whole-heart coverage would substantially advance our understanding of the mechanisms underlying flow-dependent human congenital heart disease and could facilitate earlier therapeutic intervention.

Complementary to *in vivo* flow mapping, patient-specific computational fluid dynamics (CFD) has been applied to fetal ventricular geometries reconstructed from clinical imaging. This enables spatially resolved estimates of intraventricular velocity, pressure, and wall shear stress. In addition, CFD analyses of the fetal heart indicate that shear stress and related flow parameters change substantially over gestation, including reductions in peak shear stress near the mitral valve region,^53^ highlighting the time-dependent nature of the fetal ventricular mechanotransductive milieu. Furthermore, accurate flow analysis requires patient-specific cardiac geometry. Image-based CFD driven by patient-specific 4D ultrasound data have been used to characterize fetal ventricular flow mechanics, including vortex formation and wall shear stress.^54,55^ Extending these approaches to congenital disease, CFD simulations of fetal tetralogy of Fallot demonstrate alterations in hemodynamics in the heart, including elevated diastolic shear stress in the right ventricle.^56^ Collectively, these studies emphasize that precise assessment of fetal cardiac flow demands high spatiotemporal resolution combined with anatomically accurate, patient-specific heart structures.

### C. Ventricular wall stress in myocardial development

The cyclic stretch of the ventricular wall and myocardial contraction activate mechanosensors, such as SACs and integrins, which elevate intracellular calcium and activate mechanosensitive pathways that remodel the cytoskeleton and cell adhesions during trabeculation and later compaction.^57,58^ In particular, integrins serve as key mechanosensors for myocardial development. Cardiomyocyte-specific deletion of β1 integrin in mice leads to reduced myocardial proliferation and failure of normal ventricular compaction.^59^ Similarly, mechanical activation of integrins promotes focal adhesion formation and maturation pathways in developing cardiomyocytes. Mechanical cues, such as matrix stiffening, enhance myofibril organization and contractile function via focal adhesion kinase (FAK).^60,61^ Such effects of mechanical activation via matrix stiffness on myofibril registry and sarcomere organization have also been demonstrated in *ex vivo* embryonic hearts, which undergo dramatic stiffening within the first few days of development.^62,63^ Over time, the combined effects of mechanical and biochemical cues shape the developing ventricular wall into a structured, multi-layered tissue comprising an outer compact and an inner trabecular myocardium. In summary, mechanical cues in the developing heart are fundamental in orchestrating proper morphogenesis, ensuring myocardial trabeculation, compaction, and valve formation where required.

## III. PHYSIOLOGICAL MECHANICAL STRESSES IN THE ADULT VENTRICLES

**FIG. 1.**
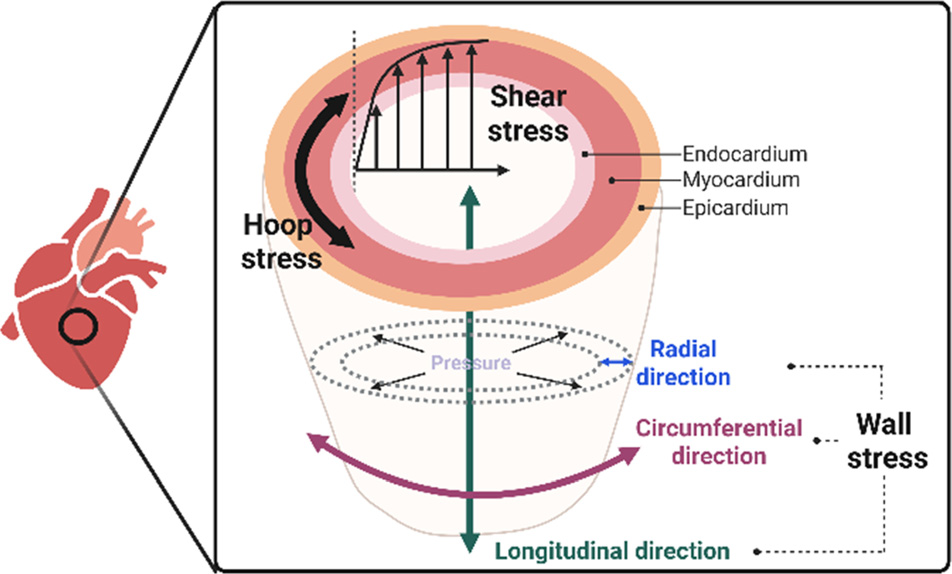
Physiological mechanical stresses in the adult heart in the radial, longitudinal, and circumferential directions.

While mechanical cues during development primarily act as morphogenetic regulators, similar mechanosensitive mechanisms in the adult heart are activated in response to physiological functional loading conditions. Myocardial wall mechanical stresses arise from intrinsic cardiac contractility, preload-induced diastolic stretch, afterload during systolic ejection, and fluid shear stress acting on the endocardium and the coronary vasculature.^64–66^ During the cardiac cycle, wall normal stresses develop within the myocardium and can be resolved into radial (transmural), longitudinal (base-to-apex), and circumferential components (Fig. 1).^67^ In addition, ventricular filling and ejection modulate intraventricular pressure, giving rise to hoop stress within the ventricular wall. Moreover, blood flow within the chambers and coronary circulation, as well as pericardial fluid motion, impose fluid shear stress on the endocardium, myocardium, and epicardium.^53^ The combination of these various stresses generates a spatiotemporally dynamic stress field within the heart, influencing cardiac function and modulating cell behavior. This section outlines the major physiological mechanical stresses in the heart, their functional implications, and the shared mechanosensors that translate these loading conditions into cellular responses.

### A. Mechanical stresses generated by intraventricular pressure

Effective cardiac function relies on coordinated mechanical interactions between the myocardium and circulating blood. Relaxation of cardiomyocytes allows ventricular chambers to fill with blood, while systolic contraction drives blood ejection.^68^ These phases generate dynamic changes in stress within the ventricular wall.^64,65^ Concurrently, blood flow induces fluid shear stress along the endocardium, the inner surface of the chamber wall.^66,69^ Several factors govern the mechanical stresses in the ventricle wall. First, elevated intraventricular pressure directly increases the magnitude of the hoop stress in the ventricular wall.^67^ This pressure also affects flow rate of blood in the heart, thereby altering the amount of fluid shear stress on the endocardium.^70^ Second, changes in chamber geometry significantly affect stress distribution. For instance, during systole, ventricular contraction alters the size and shape of the chamber, reshaping the direction and distribution of both wall stress in the myocardium and fluid shear stress on the endocardium. Notably, wall stress in the myocardial wall peaks during protosystole, the early phase of systole marked by the opening of the aortic valve, when the ventricular chamber exhibits its largest diameter and thinnest wall thickness.^71^ Finally, the cyclical nature of cardiomyocyte contraction links mechanical stress dynamics directly to heart rate. Given the viscoelastic material properties of myocardial tissue, the effects of these dynamic stresses on tissue stiffness are influenced by the contraction time rate.

Quantitatively, myocardial wall stresses in the radial, circumferential, and longitudinal directions have been estimated using both direct intramyocardial measurements and model-based approaches. In adult cat hearts, total intramyocardial stress measured using a microtransducer inserted into the myocardium and interpreted using mathematical models based on large elastic deformation theory and thick-walled assumptions yielded wall stress values of approximately 0.31–8.4 kPa in the radial direction, 4.6–16 kPa in the circumferential direction, and 2.2–3.5 kPa in the longitudinal direction.^72^ In contrast, human myocardial wall stresses estimated using carotid tonometry, Doppler imaging, and speckle-tracking echocardiography in combination with a thin-wall pressure vessel model have been reported to range from 23 to 38 kPa circumferentially and from 13 to 21 kPa longitudinally.^73^ These differences indicate that myocardial wall stress values vary substantially depending on species, measurement techniques, and the mathematical framework.

Recent reviews have further emphasized that reported wall stress values exhibit significant method-dependent variability.^74^ Calculated stress can differ according to (i) geometric assumptions, such as spherical, ellipsoidal, or image-derived 3D ventricular geometry, (ii) the imaging modality used to determine ventricular radius and wall thickness, (iii) the pressure input, such as invasive left ventricular catheter pressure or cuff systolic blood pressure as a surrogate, and (iv) the temporal definition of stress over the ejection phase. Consequently, it is important to consider these sources of variability when interpreting myocardial wall stress as a mechanical environment for cardiac cells in the context of mechanotransduction.

Several computational approaches have been utilized to analyze how intraventricular pressure and wall thickness influence myocardial wall stress. One of the most frequently used is Laplace's law, which relates the geometry of the ventricular chamber to intraventricular pressure and wall stress.^75^ The generalized form of Laplace's law for a thin cardiac wall ( $h\leq R_{\text{cir}}\text{ and }R_{\text{long}}$) is expressed as follows:^75^

$$\frac{\sigma_{\text{cir}}}{R_{\text{cir}}}+\frac{\sigma_{\text{long}}}{R_{\text{long}}}=\frac{P}{h}.$$

**FIG. 2.**
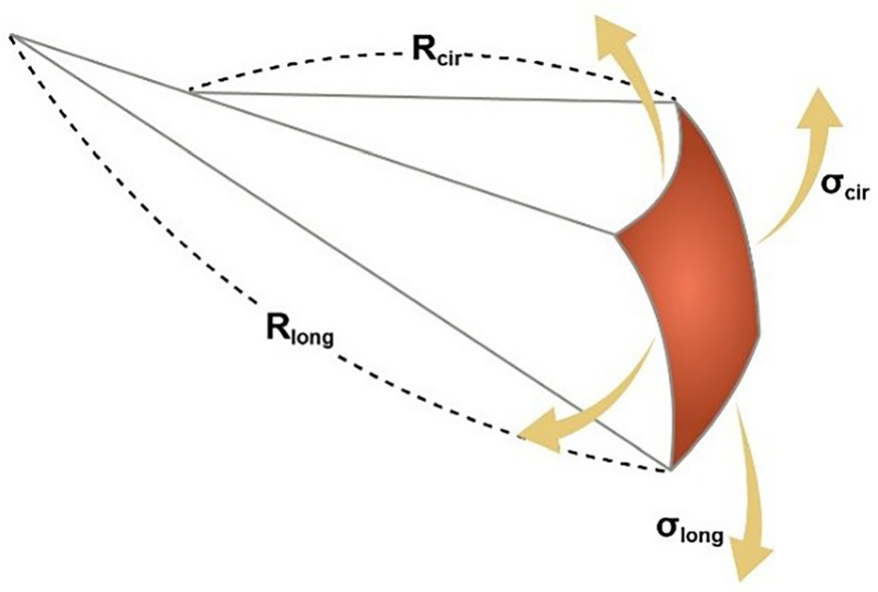
Schematic illustration of wall stresses and radii of curvature used in the generalized form of Laplace's law for the cardiac wall.

Here, the subscripts *cir* and *long* refer to the circumferential and longitudinal directions, respectively. *σ* represents wall normal stress, *R* indicates the corresponding radius of curvature of the endocardium (Fig. 2), *P* is the intraventricular pressure, and *h* is the wall thickness of the ventricular chamber. Using this equation, circumferential and longitudinal wall stresses in the ventricular wall can be calculated under the assumption that the ventricular wall is isotropic, homogeneous, and subjected to constant stress. Based on this model and other thin-wall mathematical models, the normal range for maximum wall stress in the human ventricular wall is estimated to be between 12.3 and 37.7 kPa.^73,76^ While this approach provides reasonably accurate estimates of average wall stress, it does not account for the spatial heterogeneity of stress across the ventricle wall.^65^

To address the limitations of thin-wall theories, including Laplace's law, a thick-shell theory was proposed for more accurate wall stress calculation in the left ventricle.^65^ Unlike thin-wall models, the thick-wall theory enables explanation of stress variations from the endocardium to the epicardium and incorporates the effects of radial and bending stresses.^77^ It relies on several assumptions: (1) the ventricular wall is an isotropic and homogeneous elastic material, (2) the tissue is incompressible with constant volume during the cardiac cycle, and (3) chamber geometry and intraventricular pressure are measured instantaneously throughout the cycle. According to this model, wall stress is maximal at the endocardium and decreases toward the epicardium. Moreover, bending stresses lead to a progressively larger stress gradient from the equator to the apex. Similar to thin-wall model, thick-wall elasticity and shell models assume that cardiac wall deformation occurs within a linear elastic regime, with strain limited to 5%–10%. However, echocardiographic recordings of the left ventricle in healthy individuals demonstrate that longitudinal strain ranges from −24% to −16%,^78^ indicating that myocardial deformation exceeds the linear range. To accommodate this, a thick-wall sphere ventricle model capable of capturing larger, nonlinear deformations was introduced.^79^ This model confirmed that wall stress concentrations are highest in the endocardium, compared to the myocardium and epicardium. This spatially nonuniform stress distribution may be associated with a higher susceptibility of subendocardial regions to ischemia.^80^ Despite these advancements, the model remains based on idealized geometries such as spheres or ellipsoids, which do not fully replicate the anatomical complexity and structural features of the human ventricular chambers. Moreover, cardiac tissue is anisotropic, as it is composed of aligned cells and ECM, predominantly along the longitudinal direction.^81^

To more accurately capture the complex geometry and material behavior of the cardiac chamber, finite element models (FEMs) have been applied for detailed stress analysis. These computational models incorporate nonlinear and anisotropic material properties, passive and active material parameters, fiber-oriented myocardial architecture, and large deformation mechanics, based on experimental data mostly obtained from animal models.^65,82,83^ In addition, advancements in FEMs have enabled incorporation of residual stresses and increasingly accurate representations of ventricular structure, including modeling the ventricular wall as stacked fibrous layers rather than as a uniform structure and using realistic three-dimensional geometries and fiber patterns obtained by advanced imaging techniques.^84,85^ Over time, FEMs of the human ventricle have progressed from simplified cylindrical geometries to more anatomically accurate representations, including elliptical left ventricular models, biventricular geometries, axisymmetric ventricles, anatomically detailed left ventricles, anatomically realistic biventricular models, and, most recently, whole-heart models that capture the full structural complexity of the human heart.^86^ Notably, for improved quantitative accuracy and translational relevance, there has been growing emphasis on patient-specific FEMs that integrate noninvasive imaging modalities such as MRI, enabling individualized assessment of cardiac mechanics under physiological and pathological conditions.^87^

Additionally, material properties and boundary conditions to simulate disease-specific constraints on the chamber have been introduced. For instance, Guccione and colleagues utilized FEMs to demonstrate that the Myosplint reduces myocardial stress in failing hearts with increased ventricular stiffness.^88^ Similarly, Walker *et al.* applied FEM to evaluate the effects of linear repair in left ventricular aneurysms, incorporating anisotropic aneurysmal tissue, reduced isometric tension, and septal aneurysm material parameters derived from sheep models, and showed a significant reduction in systolic wall stress in the ventricle, although septal and diastolic stresses remained elevated.^89^ These FEM-based studies offer powerful tools for quantifying regional stress and strain distributions throughout the cardiac cycle under both normal and pathological conditions.^65,90^

### B. Generation of fluid shear stress in chambers, coronary vasculature, and the pericardial space

The spatiotemporal distribution of fluid shear stress along the endocardium is influenced by several factors, including chamber geometry, the cardiac cycle, and areas of stagnant or vortex flow within the chamber.^91,92^ Wall shear stress can be quantified using various imaging and computational techniques, such as echocardiographic Doppler imaging, particle image velocimetry, or CFD.^69,93,94^ For example, using vector flow mapping with color Doppler imaging, peak left ventricular wall shear stress values, ranging from 30.74 to 53.90 Pa with an average of 41.16 Pa, were reported in the anterior wall segment during the rapid filling phase. This study also showed that reduced ventricular compliance and relaxation in aged individuals elevate wall shear stress by increasing intraventricular pressure during diastole.

More recently, advances in 4D-flow MRI have enabled acquisition of time-resolved, three-dimensional velocity fields, which provide various flow-related metrics, including kinetic energy of blood flow, viscous energy loss, and intraventricular pressure gradients using the Bernoulli equation or the Navier–Stokes equation.^95^ Although the spatial resolution of current 4D-flow MRI remains insufficient for precise quantification of wall shear stress, this technique provides important insights into intraventricular flow organization and cardiac valve-related hemodynamics.^96^ In addition, compared with 4D echocardiographic doppler imaging, 4D-flow MRI offers full three-dimensional velocity vector fields and enables derivation of a broader range of hemodynamic parameters.^94^ More recently, machine learning-based approaches have been implemented to improve ventricular segmentation during post-processing and more robust estimation of wall shear stress.^97,98^

Additionally, CFD simulations have revealed the formation of a vortex ring at the mitral valve during diastolic filling. This vortex ring facilitates effective blood mixing and supports efficient transition from diastolic inflow to systolic outflow.^35^ Beyond assessments in healthy hearts, fluid dynamics have also been investigated in pathological conditions.^99^ Patients with dilated cardiomyopathy (DCM) with an ejection fraction of approximately 18% exhibited significantly reduced vortex formation, impaired left ventricular filling, and increased flow stagnation compared to healthy subjects. Hypertrophic cardiomyopathy (HCM) patients showed cirrostratus-cloud-like vortex structures instead of the well-defined major vortex ring observed in healthy subjects.^100^

Wall shear stress generated by blood circulation affects not only the endocardium lining the chamber inner walls but also other endothelial cells in the myocardium and epicardium. In particular, shear stress in coronary vessels plays a critical role in regulating endothelial cell function, vascular remodeling, and the progression of cardiovascular diseases.^101–104^ In coronary arteries, the time-averaged shear stress over the pulsatile cycle ranges from 1 to 7 Pa, as obtained by doppler ultrasound image acquisition and CFD analyses. Shear stress in capillaries is generally less than 8 Pa.^105,106^ However, it has been reported to reach values up to approximately 100 Pa.^107^ In contrast, shear stress in veins is typically much lower than in arteries, ranging from 0.1 to 0.6 Pa.^108^ Computational simulations using anatomical data have estimated wall shear stress in coronary capillaries to be significantly higher than in other capillaries, with values ranging from 0.7 to 100 Pa.^109^ Disturbance or complete blockage of blood flow in coronary vessels can lead to myocardial ischemia or infarction, resulting in tissue damage or necrosis.^110^ Notably, pathologically elevated wall shear stress by coronary flow is associated with a higher risk of plaque erosion, plaque rupture, and calcific plaque formation.^111^

In addition to the blood within cardiac chambers and vasculature, the fluid surrounding the heart also contributes to the mechanical environment. The pericardial fluid, which fills the narrow space between the epicardium and the parietal pericardium, serves as a lubricant and cushion for the heart.^112^ This fluid is an ultrafiltrate of plasma containing proteins, electrolytes, and phospholipids,^113^ and its volume in an average adult male typically ranges from 20 to 60 ml.^114^ Pressure within the pericardial cavity increases during diastole and decreases during systole, generating a pressure gradient that drives fluid movement.^66,115^ This movement suggests that the epicardium is subjected to shear stress from the pericardial fluid flow throughout the cardiac cycle. However, quantification of the shear stresses exerted on the epicardium and pericardium requires further study.

### C. Conversion of forces to biological signals via mechanosensors

Costameres are specialized adhesion assemblies on the sarcolemma (cell membrane) of muscle cells, including cardiomyocytes.^116^ They align with Z-disks of sarcomeres and mechanically couple the subcellular contractile units to the ECM, helping distribute contractile load laterally across the cell surface and transmit into the ECM. The key components at costameres include integrins, particularly the β1 integrin in cardiomyocytes, which are linked to the actin via connectors such as talin and vinculin. Integrin-based mechanotransduction can be described as a sequence of physically coupled steps. First, integrins bind ECM ligands and are mechanically loaded by muscle contraction or by external stretch, such as a hypertensive condition. Second, talin binds integrin cytoplasmic tails and provides a direct mechanical link to filamentous actin (F-actin), positioning talin as a primary load-bearing element in integrin adhesions.^117^ Third, when talin receives tensile forces, talin rod domains can be unfolded that exposing cryptic vinculin-binding sites, enabling vinculin recruitment and strengthening of the integrin to actin linkage.^118,119^ In single-molecule measurements, talin unfolding occurs at low piconewton forces, and vinculin binding can lock talin in an unfolded conformation, providing mechanical reinforcement under load.^120^ This initiates activation of FAK, which can then drive downstream pathways, including the Ras homolog family member A (RhoA)/Rho-associated protein kinase (ROCK), actin remodeling, and YAP-TEAD activity, in mechanically stressed cardiac cells.^121–123^

Alongside integrin-based focal adhesion complexes, the dystrophin glycoprotein complex (DGC) is a key component of the cardiomyocyte costamere, mechanically linking F-actin to the ECM.^124^ Within the DGC, dystrophin binds directly to F-actin and to dystroglycan, which is embedded in the sarcolemma and connects to laminin, thereby forming a mechanical linkage between the sarcomere and the ECM. This linkage provides structural support to muscle cells and protects the sarcolemma from contraction-induced damage.^125^ In particular, dystrophin functions as a molecular spring that mitigates mechanical stress and preserves membrane stability under load. Beyond passive mechanical support, the DGC can also act as a mechanosensor. Mechanical stretch activates AMP-activated protein kinase (AMPK) via the DGC, and this regulates downstream NO signaling.^126^ Consistent with these roles, dystrophin deficiency, as observed in Duchenne muscular dystrophy (DMD), is associated with membrane fragility, impaired force transmission, dysregulation of calcium handling, and progressive DCM due to disruption of both the mechanical and signaling functions of the DGC.^127–129^

Moreover, intercalated disks (ICDs), specialized heart-specific junctional complexes, transmit mechanical forces between adjacent cardiomyocytes.^130^ Adherens junctions (AJs) and desmosomes primarily maintain structural integrity, and gap junctions (GJs) serve mainly in electrical and biochemical signaling.^131^ In particular, mechanical load increases tension across N-cadherin/catenin complexes at AJs, promoting junctional reinforcement through α-catenin/vinculin recruitment and cytoskeletal remodeling.^132,133^ In parallel, plakoglobin, which binds to both N-cadherins or desmosomal cadherins, can translocate to the nucleus of cardiomyocytes, where it interferes with β-catenin transcriptional activity under mechanical load. Meanwhile, connexin 43 (Cx43) phosphorylation regulates gap junction remodeling in response to mechanical stress.^134,135^

Mechanical forces are also transmitted directly to the nucleus through the linker of nucleoskeleton and cytoskeleton (LINC) complex, which physically couples the F-actin to the nuclear lamina.^136^ The LINC complex consists of SUN-domain proteins in the inner nuclear membrane and KASH-domain proteins in the outer nuclear membrane.^137^ Together, these proteins physically connect the cytoskeleton to the nuclear lamina and lamina-associated chromatin across the nuclear envelope, providing a direct route through which cytoskeletal tension can influence nuclear architecture and transcriptional regulation.^138^ Force-induced deformation of the nuclear lamina alters nuclear stiffness, nuclear pore permeability, and chromatin organization,^139,140^ thereby modulating YAP/TAZ nuclear localization and transcriptional activity in mechanically stressed cardiac cells.

Additionally, membrane tension generated by tensile or shear stresses directly gates mechanosensitive ion channels, including Piezo1 and the TRP family, leading to rapid calcium influx. For example, increases in membrane tension within the lipid bilayer lower the energetic barrier for Piezo1 channel opening by inducing conformational changes in its curved, dome-like structure, thereby favoring pore dilation in response to stretch or shear.^141^ In cardiac cells, calcium entry through these mechanosensitive channels activates downstream effectors, such as calcineurin-nuclear factor of activated T cells (NFAT) and CaMK signaling pathways, which regulate gene programs associated with hypertrophic growth and fibrotic remodeling.^7,142^

### D. Mechanotransduction in cardiac homeostasis

As discussed above, cardiac tissue is continuously subjected to complex, dynamic mechanical stresses. Wall stresses generated by intraventricular pressure and myocardial contraction are sensed by cardiomyocytes through the integrin complexes, dystroglycan complexes, and SACs located in the sarcolemma and transverse-tubules (T-tubules).^143,144^ Activation of these mechanosensors modulates multiple downstream processes, including calcium handling, sarcomere alignment, stretch-induced structural changes in troponin that alter calcium ion sensitivity, nuclear translocation of YAP and muscle LIM protein (MLP), and the regulation of kinases and phosphatases.^145–149^ These processes govern excitation-contraction coupling, allowing the myocardium to adjust its contractile behavior in response to changing cardiac demands and environmental conditions.

Cardiac fibroblasts and endothelial cells are also well-known mechanosensitive cells in the heart. In the heart under physiological mechanical conditions, they maintain ECM homeostasis through a balance of ECM synthesis, cross-linking, and metalloproteinase activity.^150–156^ Interestingly, when cardiac fibroblasts were subjected to tensile (positive) or compressive (negative) strain of equal magnitude, collagen III and fibronectin expression were higher under tensile loading than under compression, whereas TGF-β1 activity was similar in both conditions.^157^

Furthermore, fluid shear stress by blood flow is a critical cue for maintaining endothelial homeostasis in the heart, particularly by suppressing EndoMT. Physiological shear is detected by surface and junctional mechanosensing structures, such as the endothelial glycocalyx,^158^ and mechanosensitive channels, which together maintain flow-adaptive states.^159^ Absence of blood flow in the left ventricles of rat hearts induces fibrosis and collagen deposition in the endocardium and subendocardial layers.^160^ In the same study, human endocardial cells cultured *in vitro* without flow showed disorganized cell alignment, whereas cells exposed to 5 dyne/cm^2^ for 24 h aligned along the flow direction. The absence of flow also induced expressions of EndoMT, apoptosis, and Notch signaling. Moreover, physiological shear stress induces NO release from endothelial cells, which helps maintain an anti-inflammatory endothelial phenotype and serves as an important paracrine signal that modulates cardiomyocyte contractility.^161,162^ While many studies have investigated how abnormal mechanical environments drive cardiac cells toward pathological phenotypes, relatively little is known about how physiological mechanical stresses in the healthy adult heart contribute to cellular homeostasis and the maintenance of normal cardiac function. A deeper understanding of the specific mechanical cues required for essential cellular functions in the adult heart will be critical for preserving cardiovascular health and developing strategies for disease prevention.

## IV. HYPERTENSION AND HYPERTROPHY

Hypertension, defined as persistently elevated blood pressure exceeding 130/80 mm Hg (systolic/diastolic), induces significant structural and functional remodeling of the cardiac chambers.^163–165^ For instance, sustained pressure overload of the ventricle can lead to ventricular hypertrophy,^166,167^ characterized by cardiomyocyte enlargement with wall thickening or chamber dilation and often accompanied by arrhythmia and fibrosis. Hypertension can induce eccentric or, more commonly, concentric hypertrophy, diagnosed by a relative wall thickness greater than 0.42.^168^ These alterations can severely impair cardiac function. Hypertensive hypertrophy engages diverse mechanotransduction pathways.^169,170^

To overcome the increased resistance from elevated intraventricular pressure, various myocardial cells activate pathways through key mechanical sensors, such as integrins,^171,172^ cell junctions, and SACs. In particular, integrins transmit forces from both intracellular myosin-driven contractions and external sources from the ECM.^173^ In addition to pressure overload, pathological cardiac overload can also arise from volume overload, for example due to valvular regurgitation or other conditions that increase chamber filling and diastolic stretch.^174^ Volume overload also activates mechanotransduction pathways, including hypertrophic growth, sarcomere depletion, increased phosphorylation of Akt and GSK3β, upregulation of cardiac LIM domain protein, and transient downregulation of desmin.^175^ In this section, we introduce several key mechanotransduction pathways involved in hypertensive cardiac hypertrophy through these cell-cell and cell-ECM interactions.

### A. RhoA-mediated cytoskeletal reinforcement

**FIG. 3.**
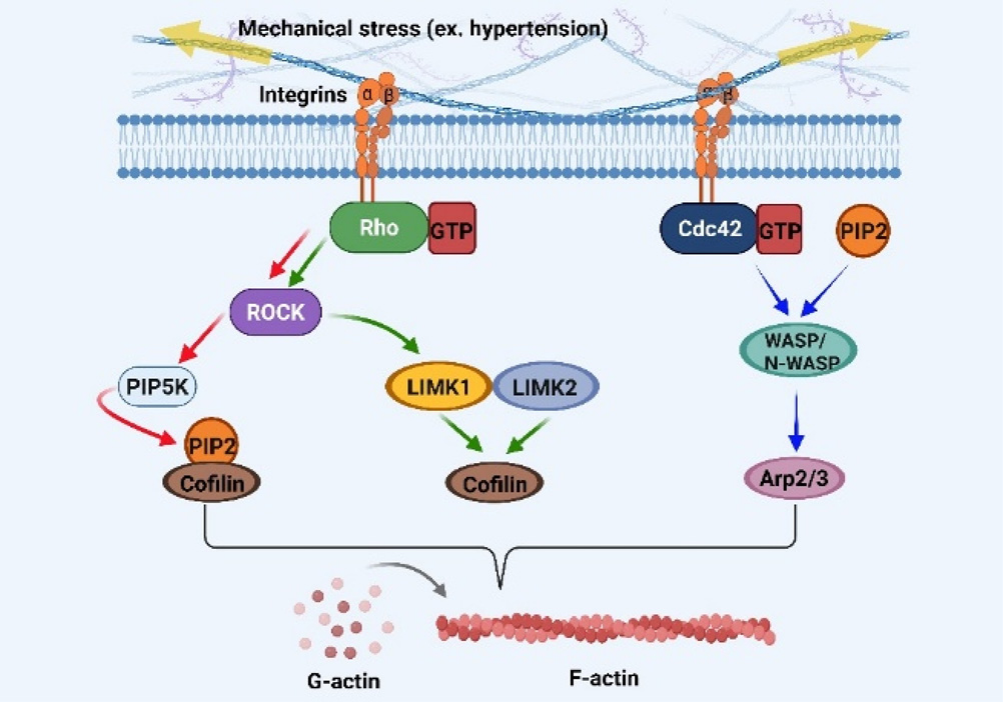
The role of RhoA/ROCK signaling in regulating actin cytoskeleton dynamics in response to mechanical stress. Upon mechanical stress, integrins activate RhoA, which then triggers several downstream pathways. First (red arrows), RhoA activates ROCK, which increases PIP5K activity, leading to elevated PIP2 production. PIP2 interacts with actin-binding proteins such as cofilin and N-WASP to stabilize filamentous actin (F-actin) and inhibit cofilin-mediated actin filament severing. Second (green arrows), ROCK phosphorylates LIMK1 and LIMK2, which inactivate cofilin, further preventing actin depolymerization and promoting stress fiber formation. Finally (blue arrows), PIP2 activates N-WASP, which facilitates the activation of the Arp2/3 complex, promoting actin nucleation and elongation. Together, these pathways coordinate the polymerization of globular actin (G-actin) into F-actin to maintain cytoskeletal stability or to induce hypertrophy in response to mechanical stress.

When mechanical load is increased under hypertensive conditions, integrin signaling through FAK is well known to activate the RhoA/ROCK-Phosphatidylinositol 4-phosphate 5-kinase (PIP5K) signaling cascade that promotes cardiomyocyte growth.^135,176^ This pathway is essential for cytoskeletal reorganization, enhancement of mechanosensitivity, and activation of downstream effectors, including ROCK and PIP5K (Fig. 3).^177–179^ Specifically, RhoA-induced activation of ROCK promotes stress fiber formation, microtubule stabilization, and actin polymerization,^180–184^ thereby reinforcing cell integrity and protecting cardiac cells from load-induced damage. A key component of this response is ROCK-driven activation of PIP5K, which increases phosphatidylinositol 4,5-bisphosphate (PIP2) synthesis.

PIP2 directly regulates actin dynamics by binding to actin-associated proteins. Cofilin, which enhances actin filament depolarization, is inhibited by binding to PIP2.^185^ In addition, PIP2 binds to the actin-capping protein CapZ, reducing its affinity for the barbed ends of actin filament.^186^ This permits excessive filament elongation, leading to disorganized sarcomere architecture and the initiation of pathological cardiac hypertrophy in cardiomyocytes.^187–190^ Consistent with this, treatment with the RhoA inhibitor C3 transferase, the ROCK inhibitor Y27632, or the PIP2 scavenger neomycin inhibited actin filament formation, even in the presence of mechanical tension.^191^

Notably, the neuronal Wiskott-Aldrich syndrome protein (N-WASP)-actin-related protein 2/3 complex (Arp2/3) pathway activated by PIP2 or cell division cycle 42 (Cdc42) functions independently of ROCK to regulate actin polymerization.^191,192^ These interactions facilitate the Arp2/3-mediated actin nucleation, thereby maintaining cytoskeletal integrity (Fig. 3).^135,193,194^ In parallel, RhoA/ROCK-dependent actin reorganization can be obtained by phosphorylating LIM kinase 1 (LIMK1) and LIM Kinase 2 (LIMK2), which inactivate cofilin and suppress actin filament severing.^178,184,195–197^ Collectively, these actin-related mechanotransduction pathways represent an important driver of cardiac hypertrophy, particularly through increased phosphorylation of FAK and PIP2 localization at the Z-disk of sarcomeres, where actin assembly occurs.^198^

RhoA signaling also serves as a critical upstream regulator that coordinates cytoskeletal and hypertrophic responses through serum response factor (SRF) and myocardin-related transcription factor A (MRTF-A).^199^ The formation of actin filaments by RhoA activation frees MRTF-A to translocate to the nucleus, triggering the expression of SRF target genes.^200^ SRF is known to induce the expression of hypertrophic genes, including atrial natriuretic factor, brain natriuretic peptide (BNP), and α-myosin heavy chain (MYH), in studies using neonatal rat ventricular cardiomyocytes (NRVMs) and SRF-floxed transgenic mice.^201^ Additionally, hypertrophic stimuli such as phenylephrine robustly induce SRF-mediated transcription. This is further confirmed by the loss of SRF via Cre-recombinase, which results in a significant inhibition of hypertrophic gene expression. These results indicate a critical function of SRF activated by RhoA in cardiac hypertrophy, which can develop in response to abnormal mechanical loads through RhoA and ROCK.

Many studies on cardiac mechanotransduction, including those cited in this review, have utilized NRVMs. In particular, NRVMs offer several experimental advantages compared to adult cardiomyocytes, including lower cost and greater ease of cell isolation.^202^ However, it is well established that NRVMs differ from adult cardiomyocytes in several key features, including sensitivity to drugs that affect the non-sarcomeric cytoskeleton,^203^ responses to hypoxia,^204^ proliferative capacity,^205^ and excitation-contraction coupling.^206^ With respect to RhoA, which is discussed in this section, overexpression of RhoA induces apoptosis in NRVMs, whereas in adult cardiomyocytes it does not significantly alter cardiac structure or contractile function.^199,207^ These observations suggest that mechanotransductive responses may be age-dependent and should be interpreted with caution when extrapolating findings across developmental stages.

### B. Mitogen-activated protein kinase (MAPK)-mediated mechanotransduction

A hypertensive environment or mechanical stretch also induces extracellular signal-regulated kinases (ERKs) signaling and hypertrophy.^208^ The core of this pathway is a three-tiered phosphorylation cascade comprising MAP3K and MAP2K.^209^ Within this pathway, MAPK subfamilies, including ERK1/2, Jun N-terminal kinase (JNK), and p38 MAPKs, have distinct roles.

Pressure overload activates ERK1/2 via angiotensin II, which exaggerates cardiac hypertrophy and increases apoptosis.^210–212^ In particular, ERK1/2 activation promoted concentric hypertrophy, while inhibition of ERK1/2 through genetic deletion or inhibitors led to eccentric hypertrophy. Similarly, in engineered heart tissue models exposed to cyclic stretch, ERK1/2 activation resulted in greater cell width, whereas its inhibition caused cell lengthening. Interestingly, when ERK1/2 activation is almost completely inhibited, cardiac hypertrophy still occurs in response to mechanical stress.

The JNK pathway is also mechanosensitive, primarily due to its ability to phosphorylate transcription factors involved in hypertrophy and stress responses.^209,213^ A 20% mechanical strain applied to NRVMs strongly activated the JNK pathway and upregulated hypertrophic genes.^210,214^ This stretch-induced JNK activation exceeded the effects of angiotensin II without stretch, indicating that mechanical stress alone can directly activate the stress kinase cascade. Additionally, inhibition of calcineurin attenuated cardiac hypertrophy via JNK by interrupting intracellular calcium signaling during pressure overload.^215^ This calcineurin-mediated hypertrophy was independent of ERK1/2 and p38 activation. In addition, JNK activation under pressure overload is MEKK1-dependent, but cardiac hypertrophy still occurs in MEKK1-/- mice.^216^ This suggests the existence of bypass mechanisms that either activate JNK independently of MEKK1 or induce hypertrophy without JNK involvement.

The p38 MAPK pathway also contributes to cardiomyocyte hypertrophy induced by mechanical stress in a time-dependent manner.^209,213^ For instance, when cardiomyocytes were stretched, the p38 pathway was activated independently of humoral factors like angiotensin II or endothelin-1.^217^ This activation, mediated by the integrin-FAK signaling axis, peaked within 15 min of stretching and diminished after 60 min. Treatment with the selective p38 MAPK inhibitor SB202190 confirmed its role in the stretch-induced synthesis of hypertrophic factors. Similarly, the p38 MAPK pathway was activated in transverse aortic constriction (TAC) models via GRB2-FAK interactions.^218^ Notably, experiments with dominant-negative p38α and p38β mutants revealed that while cardiac hypertrophy can occur without p38 MAPK activation, fibrosis is significantly reduced.^219^ Taken together, these findings indicate that cardiac hypertrophy is not driven by a single MAPK pathway, but rather arises from the interplay of multiple interconnected signaling mediators.

### C. Hypertrophic remodeling driven by YAP/TAZ

YAP/TAZ are well-known mechanosensitive factors and are also involved in hypertensive hypertrophy.^220,221^ Hypertension, ECM stiffness, and cell contractility can all influence the activity and localization of YAP/TAZ,^222,223^ driving diverse cell responses, including proliferation, cytoskeletal remodeling, cellular energy homeostasis, and metabolism adaptation.^224,225^ The role of YAP in mediating cardiac hypertrophy under pressure overload has been extensively investigated. For instance, inhibition of YAP1 significantly attenuated hypertrophy and mitochondrial damage in chronic pressure overload.^226^ Conversely, YAP1 overexpression via tamoxifen injection replicated the hypertensive hypertrophy, and YAP1 activation perturbed mitochondrial function by repressing dynamin-related protein 1 (DRP1) and mitofusin-1 (MFN1). In a TAC mouse model, YAP activation promotes aerobic glycolysis by upregulating glucose transporter 1 (GLUT1) through interactions with TEAD and hypoxia-inducible factor 1α (HIF-1α), thereby facilitating the accumulation of metabolic intermediates.^227^ In contrast, cardiac-specific YAP deficiency (YAPch-KO) leads to impaired glycolysis, reduced hypertrophy, and exacerbated heart failure under pressure overload. These studies demonstrate a critical role of YAP-mediated metabolic adaptation in responding to increased mechanical stress.

### D. Mechanosensitive calcium signaling

SACs are mostly nonselective cation channels and open in response to mechanical deformation.^228,229^ This permits Ca^2+^ influx, triggering calcium-induced calcium release in cardiomyocytes.^230^ In response to this, calcineurin drives myocardial hypertrophy by dephosphorylating NFAT transcription factors, enabling their nuclear translocation to regulate gene expression.^231,232^ In particular, pressure overload significantly increased calcineurin activity and NFAT2 dephosphorylation, indicating its involvement in hypertrophic signaling.^233^ Calcium-mediated mechanotransduction that promotes hypertrophy may also intersect with patient-specific genetic mutations. In particular, mutations in MYBPC3 and MYH7, which are well-established causes of HCM,^234^ are associated with contractile dysfunction, altered myofilament Ca^2+^ sensitivity, and abnormal intracellular calcium homeostasis.^235^ These alterations can enhance Ca^2+^-dependent hypertrophic signaling, including activation of the calcineurin-NFAT axis.^236^ The impact of such gene-specific perturbations highlights the importance of personalized therapeutic strategies in managing mechanically driven cardiac hypertrophy.

Among SACs, Piezo1, which exhibits a slight preference for Ca^2+^ over Na^+^, K^+^, and Mg^2+^,^237^ has been found to be significantly upregulated under pressure overload.^130^ NRVMs showed that activation of Piezo1 by mechanical stretching or the agonist Yoda1 induces hypertrophy, as evidenced by increased cell size and upregulation of hypertrophic genes such as atrial natriuretic peptide (ANP) and BNP. Piezo1-mediated mechanotransduction is upregulated in both rodent and human diseased hearts, demonstrating conservation of this pathway across species.^238^ However, direct comparisons of signaling profiles between NRVMs and human induced pluripotent stem cell (iPSC)-derived cardiomyocytes reveal species-specific differences in the expression patterns of cardiac G protein-coupled receptors and downstream effector expression, underscoring the importance of human cardiac models for therapeutic translation.^239^

TRP channels are nonselective, calcium-permeable cation channels that mediate calcium influx and contribute to cardiac hypertrophy.^240^ TRPV4 activation enhances calcium influx, amplifies calcium release through ryanodine receptors (RyR), and exacerbates calcium overload in cardiomyocytes.^241–243^ In addition, physiological stretch activates NOX2-dependent ROS (X-ROS) production, which sensitizes cardiac-specific RyR2 in a stretch-dependent manner, triggering a burst of Ca^2+^ sparks.^244,245^ These effects increase contractility in the short term but lead to calcium dysregulation, arrhythmogenic Ca^2+^ waves, cellular damage, and impaired cardiac function over time. Moreover, overexpression of TRPC3/6 showed enhanced calcineurin-NFAT signaling, leading to enlarged hearts under pressure overload.^246–248^ These results collectively highlight the pivotal role of calcineurin-NFAT signaling, which can be activated by SACs, in the progression of pathological hypertrophy.

Titin has also been proposed as a key sarcomeric mechanosensor that couples sarcomeric stress and strain to calcineurin-NFAT signaling.^249,250^ Under diastolic stretch, the extensible I-band regions undergo conformational transitions that tune titin-based stiffness, and PEVK-actin interactions can introduce a viscous resistance that opposes filament sliding and contributes to stretch-history-dependent passive force.^251^ Mechanistically, the Z-disk of titin interacts with MLP, which facilitates the localization of calcineurin to the Z-disk and is known to play an important role in activation of the pro-hypertrophic calcineurin-NFAT signaling pathway.^252^ In addition, MLP has been reported to translocate directly to the nucleus and modulate hypertrophy-associated gene expression.^253^

## V. CARDIAC FIBROSIS

**FIG. 4.**
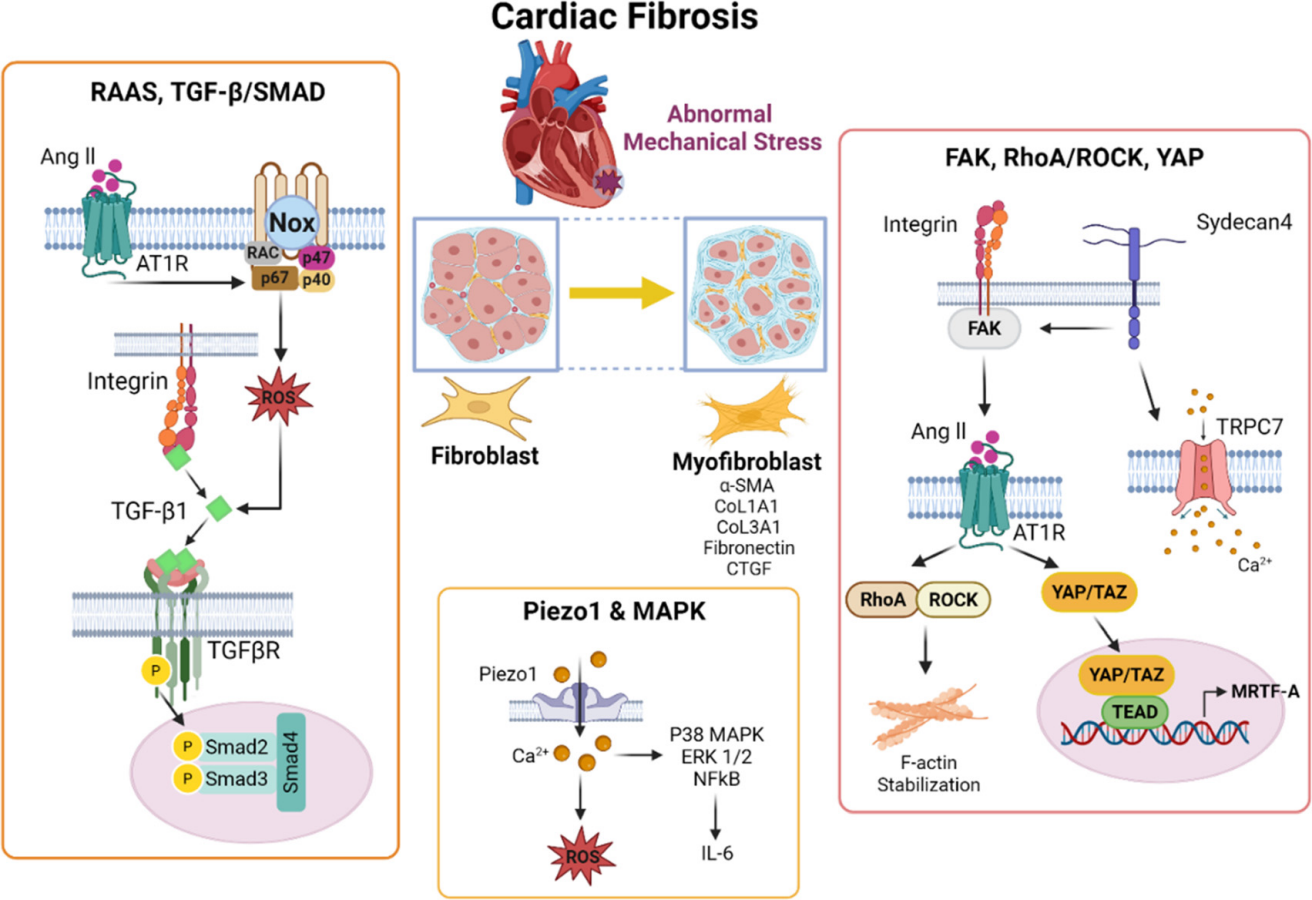
Mechanotransduction pathways linking pathological mechanical cues and hypertensive load to the initiation and progression of cardiac fibrosis.

Hypertension causes not only hypertrophy but also fibrosis in the heart.^254^ Cardiac fibrosis is characterized by excessive ECM deposition, increased myocardial stiffness, and impaired cardiac function.^254–256^ Prolonged or abnormal mechanical stress, such as a hypertensive environment, activates cardiac fibroblasts, which subsequently transdifferentiate into a distinct cell type known as myofibroblasts.^255,256^ These myofibroblasts secrete elevated levels of ECM proteins, including fibrillar collagens and fibronectin.^257,258^ Key mechanosensitive mediators such as TGF-β and YAP exacerbate this fibroblast transdifferentiation process, thereby compromising both diastolic relaxation and systolic contraction.^259^ Moreover, this maladaptive remodeling establishes a positive feedback loop in which a stiffened, fibrotic environment further activates cardiac fibroblasts, driving excessive ECM deposition and matrix stiffening. This ultimately reduces myocardial compliance and, in severe cases, progresses to heart failure. As introduced in Sec. III, cardiac cells can respond to mechanical stress and environmental changes through various sensors (Fig. 4), including integrins, cytoskeletal factors, nuclear envelope components, and SACs.^260^ In this section, we introduce key mechanotransduction pathways involved in the development and progression of cardiac fibrosis.

### A. TGF-β/SMAD signaling axis

One of the well-known signaling pathways that plays a pivotal role in the progression of cardiac fibrosis is the TGF-β/suppressor of mothers against decapentaplegic (SMAD) pathway.^261^ Mechanical stress releases active TGF-β1 from its latent complex stored in the ECM through traction forces transmitted by integrins binding to the latency associated peptide,^262,263^ thereby enabling active TGF-β1 to bind its receptors. This activation leads to an increase in phospho-SMAD2/3 levels and fibrosis progression.^264^ In particular, receptor-activated SMADs (R-SMADs), especially SMAD3, have been shown to regulate key fibrosis-related genes, including alpha-smooth muscle actin (α-SMA), type I collagens (COL1A1 & 2), COL3A1, fibronectin, and connective tissue growth factor (CTGF), driving fibroblast-to-myofibroblast transdifferentiation. In TAC models, SMAD3 deletion significantly reduced fibrosis, as demonstrated by decreased collagen deposition and ECM-related gene expression, highlighting the role of TGF-β-mediated fibrosis in response to pressure overload.^261,265,266^ Conversely, SMAD2 deletion had relatively less pronounced effect, confirming SMAD3 as the primary effector of TGF-β-driven fibrosis.

### B. FAK-mediated mechanosensing and fibrosis

Integrin-FAK signaling is central to the progression of cardiac fibrosis in response to mechanical cues.^267^ Through FAK activation, integrins sense mechanical changes in the surrounding ECM and initiate downstream pathways, such as RhoA/ROCK, MAPK,^268^ and YAP, driving fibroblast activation and ECM remodeling.^269^ Under pathological conditions, integrins serve as key mediators that sense the abnormal mechanical cues and induce cardiac fibrosis. αv integrins bind ECM ligands to activate not only latent TGF-β but also FAK/SRC signaling, promoting fibroblast proliferation, migration, and transdifferentiation into myofibroblasts.^267^ Moreover, increased expression of αvβ3 and αvβ5 integrins enhances myofibroblast transdifferentiation,^219,270^ scar expansion, and contractile dysfunction all of which could be reversed by treatment with the αv integrin inhibitor cilengitide. More recently, the focal adhesion-associated kinase, SRC, was identified as a key mediator that enables fibroblasts to detect matrix stiffness via integrins, and combined inhibition of SRC and TGF-β synergistically reduced cardiac fibrosis.^271^ These observations highlight that not only TGF-β but also integrin-FAK-SRC signaling must be considered together for more effective therapeutic intervention targeting mechanotransduction-mediated cardiac fibrosis.

In coordination with integrins, syndecan-4 facilitates focal adhesion assembly and downstream FAK activation, thereby promoting cardiac fibroblast activation and ECM remodeling in response to mechanical stimuli.^272^ Several studies have demonstrated that syndecan-4 regulates the coupling of vinculin to F-actin, ensuring proper cytoskeletal organization,^273^ and mechanotransduction.^260^ In particular, with respect to fibrosis-related mechanical signaling, syndecan-4 knockout mice subjected to pressure overload, exhibited significantly reduced expression of collagen I/III and myofibroblast markers.^274^ Interestingly, this impaired collagen expression was reduced by treatment with a calcineurin inhibitor and NFAT blockers, showing that syndecan-4 regulates fibroblast activation and ECM production through the calcineurin/NFAT signaling pathway.^141,275^ Collectively, these analyses imply that the integrin and syndecan-4 signaling pathways are central mechanotransduction networks in cardiac fibrosis progression.

### C. Rho/ROCK-driven fibroblast activation and fibrosis

The Rho/ROCK signaling pathway, which is closely linked to integrin-FAK signaling, is elevated in the myocardium under hypertension and plays a pivotal role in hypertension-associated cardiac fibrosis.^276,277^ The ROCK inhibitor Y-27632 treatment or Rho-kinase inhibition have been shown to effectively mitigate inflammation and cardiac fibrosis during hypertensive cardiac remodeling.^278–280^ Particularly, Rho/ROCK signaling has been increasingly recognized as a key downstream mediator of TGF-β signaling in the context of myofibroblast transdifferentiation and fibrosis.^281^ Studies using human fibroblasts demonstrated that TGF-β1 stimulation significantly upregulated RhoA and α-SMA, a key marker of fibroblast activation, and promoted reinforcement of vinculin-enriched focal adhesions.^282^ In TGF-β1-stimulated cardiomyocytes, treatment with Y-27632 suppressed profibrotic marker expression by inhibiting downstream PKC-δ and SMAD3 signaling pathways.^283^ Moreover, Rho/ROCK signaling has been identified as a downstream component of non-SMAD pathways activated by TGF-β, contributing to epithelial-to-mesenchymal transition (EMT) in epithelial cells.^284,285^ These findings indicate that disrupting Rho/ROCK signaling can mitigate TGF-β-mediated fibroblast activation and fibrosis, which may apply to cardiac fibrosis driven by hypertension and its promising therapeutic avenue.

### D. YAP-controlled fibrotic remodeling

In parallel with the hypertrophic responses observed in cardiomyocytes under hypertensive conditions, YAP/TAZ signaling in cardiac fibroblasts has emerged as a central mediator of fibrosis in response to mechanical stress, including hypertension and changes in ECM stiffness.^220^ Experiments comparing cardiac fibroblasts isolated from aortocaval fistula (ACF) hearts and sham controls revealed that softer substrates (2 kPa) attenuated YAP nuclear translocation and suppressed profibrotic gene expression,^187^ whereas pathologically stiffer substrates (25 and 50 kPa) enhanced YAP activation and the expression of profibrotic markers. Recently, myofibroblasts seeded onto a photoresponsive hydrogel exhibited reduced substrate stiffness from stiff (28 kPa) to soft (8 kPa) using 365 nm UV light. In the presence of a TGF-β inhibitor, this softening synergistically decreased expression of α-SMA and reduced nuclear YAP1.^271^ In cardiac fibroblasts, YAP/TAZ activity is generally pro-fibrotic. Pharmacologic YAP-TEAD inhibition (e.g., verteporfin) or fibroblast-specific YAP/TAZ deletion reduces myofibroblast transdifferentiation and ECM accumulation.^286,287^ In contrast, YAP/TAZ also exerts immunomodulatory role in the epicardium. Epicardial YAP/TAZ deletion exacerbates pericardial inflammation and diffuse myocardial fibrosis in the post-MI.^288^ Mechanistically, epicardial YAP/TAZ deletion is associated with reduced IFN-γ expression and fewer suppressive T-regulatory cells. These findings indicate that YAP/TAZ plays dual roles in the heart, promoting fibroblast activation in one context while restraining inflammation-driven fibrosis in another. The apparent discrepancy therefore reflects cell-type-specific functions of YAP/TAZ in cardiac mechanotransduction.

This mechano-activation of YAP can be achieved redundantly by multiple upstream signals and can simultaneously mediate several related signaling axes. For example, in addition to direct mechanical cues, YAP activation can be induced indirectly via angiotensin II,^289^ which can in turn regulate MRTF-A through TEAD-binding motifs.^290–292^ These studies collectively indicate that mechanosensitive YAP signaling plays a central role in coordinating the pro-fibrotic response via crosstalk with a number of signaling pathways, thus representing a key therapeutic target for cardiac fibrosis.

### E. SACs and MAPK in load-induced fibrotic responses

Piezo1 creates a feed-forward loop that exacerbates profibrotic signaling during pressure overload-induced cardiac fibrosis.^293–295^ Upon activation by mechanical stretch or increased ECM stiffness, Piezo1 triggers intracellular calcium signaling and reactive oxygen species (ROS) production, thereby promoting myofibroblast transdifferentiation and excessive ECM deposition.^138,238,296^ In a TAC model, cardiac-specific deletion of Piezo1 significantly attenuated fibrosis, as indicated by reduced fibrotic area and downregulation of fibrosis-related genes such as COL1A1 and COL3A1. Piezo1-mediated fibrosis and hypertrophy are driven through calcium-dependent pathways, including calcineurin and calpain, and are also modulated by the p38 MAPK pathway and the proinflammatory cytokine interleukin-6 (IL-6).^297^ Piezo1-induced IL-6 secretion further amplified this fibrotic signaling through signal transducer and activator of transcription 3 (STAT3) signaling and VEGF expression.^298–300^ In human atrial fibroblasts, Piezo1 knockdown impaired matrix stiffness sensing, while its overexpression promoted actin cytoskeleton reorganization, increased cell stiffness, and enhanced alignment.^296,301^ Piezo1 is broadly expressed and mediates mechanotransduction in multiple non-cardiac tissues, including vascular endothelium and immune cells.^302,303^ Therefore, before considering Piezo1 as a viable therapeutic target, it is essential to determine whether drug concentrations that effectively suppress cardiac fibroblast transdifferentiation and excessive ECM secretion are nontoxic to other Piezo1-expressing systems, particularly vascular and immune cells.^304^ *In vivo* validation using TAC or MI models would be required to assess whether Piezo1 targeting not only reduces fibrosis and preserves ejection fraction, but also avoids potential off-target effects, including edema, altered blood pressure, and dysregulated immune responses. An alternative or complementary strategy may involve identifying cardiac-specific downstream effectors of Piezo1-TRPM4-CAMKll signaling axis to minimize systemic side effects.^305^

Beyond Piezo1, mechanical stretch independently activates cardiac fibroblasts, inducing the expression and secretion of proinflammatory chemokines such as monocyte chemoattractant protein (MCP) 1 and 3 in a stretch-intensity-dependent manner.^301,306^ These chemokines recruit inflammatory cells, thereby exacerbating fibrosis. This stretch-induced fibroblast activation involves phosphorylation of key signaling mediators, including p38 MAPK, ERK1/2, and nuclear factor-kappa B (NF-κB). For example, inhibition of the p38 MAPK pathway using its inhibitor BIRB796 effectively mitigated cardiac fibrosis with reduced myofibroblast activation and downregulated fibrosis markers such as galectin-3 (Gal-3) and BNP under pressure overload.^307,308^

### F. RAAS in pressure-driven cardiac remodeling

The RAAS serves as both a direct and indirect modulator of cardiac fibrosis.^309^ While RAAS is classically recognized for controlling blood pressure and fluid balance, it also directly contributes to pathological remodeling of the heart.^310^ Angiotensin II promotes profibrotic responses primarily through the angiotensin II type 1 receptor (AT1R), initiating the TGF-β/Smad cascade.^311–313^ In hypertension, pressure overload activates the classical RAAS signaling, accelerating the development of cardiac fibrosis and hypertrophy through the Renin/angiotensin-converting enzyme (ACE)/AT1R axis.^314^ In a murine angiotensin II infusion-induced hypertension model, inhibition of RAAS downstream signaling using the SGK1 inhibitor decreased collagen deposition and IL-1β secretion by blocking NLRP3 inflammasome activation and reducing myofibroblast differentiation.^315^ In a rat model of pressure overload, treatment with the ACE inhibitor captopril effectively prevented pathological hypertrophy and fibrotic remodeling, with improvements including normalized blood pressure, reduced cardiomyocyte size, and restoration of the physiological myocyte to collagen ratio.^316^ In addition, in a rabbit model of right ventricular pressure overload, treatment with the AT1R blocker significantly attenuated biventricular fibrosis and apoptosis, primarily by suppressing profibrotic signaling pathways such as TGF-β1, CTGF, and RhoA/Smad2/3 signaling pathways.^317^ These findings support the efficacy of RAAS inhibitors in attenuating cardiac fibrosis under hypertensive conditions by reducing mechanical stress and directly inhibiting mechanosensitive fibrotic signaling pathways.

## VI. MECHANOSENSITIVE microRNAs IN THE HEART

**FIG. 5.**
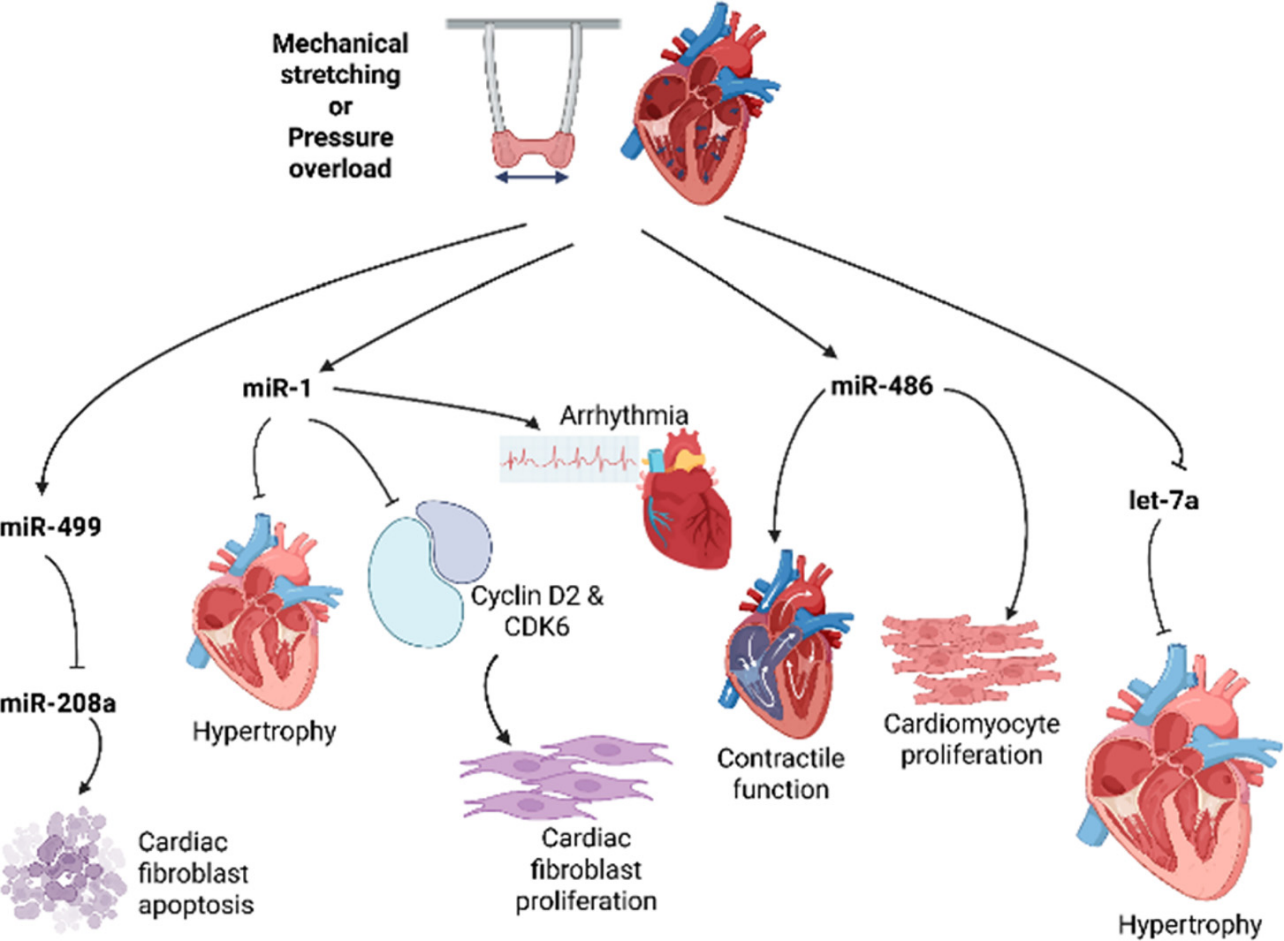
Effects of mechanical stretching and pressure overload on various microRNAs and the resulting pathways involved in cardiac function, rhythm, hypertrophy, and fibrosis.

microRNAs, or miRNAs (miRs), are non-coding RNAs that usually negatively regulate gene expression by promoting mRNA degradation or repressing mRNA translation.^318^ Some miRNAs respond to mechanical stimuli such as pressure overload or fluid shear stress. In the heart, miRNAs are involved with cardiac function, development, and heart diseases, including cardiac remodeling and heart failure. In addition to the signaling pathways mediated by coding genes discussed in Secs. III and IV, cardiac miRNAs have regulatory effects in cardiac hypertrophy, fibrosis, and apoptosis (Fig. 5).^319,320^ To gain a comprehensive understanding of these pathological processes, the precise roles of these miRNAs, many of which are closely integrated with established signaling pathways, are under continuous investigation in a variety of *in vivo* and *in vitro* disease models. In this section, we will introduce some key miRNAs in the heart, with particular attention to those responsive to mechanical cues.

As discussed in Sec. III and IV, elevated mechanical stress, such as pressure overload resulting from hypertension, can initiate pathological cardiac remodeling, including hypertrophy and fibrosis,^320,321^ processes regulated in part by miRNAs. A genome-wide study investigated gene expression changes in cultured NRVMs subjected to mechanical stretch. miR-130b was the only miRNA showing altered expression after 1 h of stretch, while eight miRNAs, including let-7f, were dysregulated after 4 h. After 24 and 48 h of stretch, 51 and 63 miRNAs, respectively, showed differential expression. Correlation analyses between dysregulated miRNAs and mRNA expression profiles also revealed that the let-7 miRNA family (let-7a, let-7c, and let-7f) had the most experimentally verified target mRNAs related to hypertrophic responses. Consistent with this, overexpression of let-7a attenuated angiotensin II-induced increases in cell size and suppressed calmodulin expression *in vitro*.^322^ *In vivo* studies using a mouse model of angiotensin II-induced cardiac hypertrophy, in which lentivirus vectors expressing let-7a were injected into the left ventricle, further confirmed the anti-hypertrophic roles of let-7a. Further studies are needed to elucidate how the let-7 family functionally interacts with other genes in regulating cardiac hypertrophy induced by mechanical stress.

miR-1, which is mainly expressed in skeletal and cardiac muscle cells, has been shown to respond to mechanical stimuli such as hypertension. It has also been shown to play a key role in controlling cardiomyocyte growth, cell fate, and rhythm.^318,323^ When cardiac hypertrophy was induced by pressure overload in TAC models, inducing miR-1 expression reversed hypertrophy and prevented maladaptive cardiac remodeling.^324,325^ In addition, miR-1 reduced Fibulin-2, an ECM protein highly expressed in developing hearts, as well as the profibrotic genes TGFB1 and CTGF. Moreover, it was shown using angiotensin II-induced cardiac fibrosis rat models that elevated miR-1 expression inhibited cyclin D2 and cyclin-dependent kinase 6 (CDK6) of cardiac fibroblasts, thus reducing proliferation. Unlike miR-1, increased expression of the miR302/367 cluster increases cardiomyocyte proliferation by repressing the Hippo signaling pathway.^326^ Additionally, miR-1 is a proarrhythmic factor, as injection of miR-1 into the infarcted myocardium has been shown to promote ischemic arrhythmias.^327,328^

miR-133 is another abundantly expressed miRNA in the heart and skeletal muscle, similar to miR-1^321^ and is also related to both hypertrophy and fibrosis. In a TAC model of cardiac hypertrophy, expression levels of miR-133 and miR-1 were reduced, more prominently in the atria and left ventricle.^329^ Another study used transgenic expression of miR-133a to prevent TAC-associated miR-133a downregulation.^31,325^ In these pressure-overloaded hearts, this improved diastolic function, reduced myocardial fibrosis, and decreased cardiomyocyte apoptosis while not affecting the extent of hypertrophy. In rat hearts with angiotensin II-dependent hypertension, miR-133a targets COL1A1, and miR-29b downregulates cardiac fibrosis.^330^ Overall, miR-133 is a key regulator of cardiac hypertrophy and fibrosis through mechanotransduction involving ECM remodeling under elevated pressure loading.^327,331^ Furthermore, miR-133 is known to be involved in cardiogenesis by targeting nuclear factor negative elongation factor A/Wolf-Hirschhorn syndrome candidate 2 (Nelf-A/WHSC2).

Some mechanosensitive miRNAs have been shown to regulate the expression of one another in cardiac cells. For instance, in human cardiac fibroblasts isolated from the adult atrial tissue (HCF-aa), mechanical stretching led to a transient increase in miR-208a expression within 0.5–2 h, followed by upregulation of miR-499 expression in 2–8 h.^332^ The regulatory roles of miR-208a and miR-499 on B-cell lymphoma 2 (BCL2), a key apoptosis regulator, were investigated in both stretched fibroblasts and a rat model of volume overload-induced heart failure. Notably, miR-499 overexpression substantially reduced miR-208a levels in the HCF-aa that underwent 1 h of stretch. 1 h of stretching significantly increased pri-miR-208a luciferase activity in the HCF-aa, which was suppressed by pretreatment with miR-499. These findings indicate that mechanical stretch induces miR-499, which represses miR-208a expression and consequently limits cardiac fibroblast apoptosis.

As discussed in Sec. II, mechanical cues generated by rhythmic cardiac contractions play a critical role during cardiac development. Disruptions in these signals, which are transduced in part by mechanosensitive miRNAs such as miR-486, have been implicated in congenital heart defects, including hypoplastic left heart syndrome (HLHS).^333^ Lange *et al.* demonstrated that miR-486 is upregulated by stretch in multiple models, including *in vitro* embryonic mouse cardiomyocytes, *in vivo* shunted sheep right ventricles (RVs), and HLHS patient RVs. Identified as stretch-responsive miRNA, miR-486 increases cardiomyocyte proliferation and ventricular growth *in vivo*. It was also shown that miR-486 improved the contractile function of embryonic mouse cardiomyocytes. Consistent with these findings, treatment of newborn mice with a miR-486 mimic led to increased left ventricle size and cardiomyocyte proliferation *in vivo*, which are closely associated with HLHS, and increased cardiomyocyte contractility *in vitro*. Finally, some miRNAs, including miR-21, have been shown to play key roles in preserving “mechanical memory” of mesenchymal cells.^334^ Similar concepts are likely to apply to cardiac fibroblasts, given miR-21's well-characterized regulation of ERK-MAP kinase signaling in pressure overload-induced fibrosis and heart failure.^335^ Taken together, these studies demonstrate that mechanosensitive miRNAs are key regulators of cardiac hypertrophy, fibrosis, apoptosis, and cardiac development by converting mechanical stimuli into coordinated signaling responses.

## VII. CONCLUSIONS

Mechanical environments, including wall and shear stresses and tissue stiffness, shape cardiac phenotypes from development through adulthood. Abnormal cues activate maladaptive programs that culminate in hypertrophy and fibrosis. However, therapeutic translation remains challenging because many distinct mechanosensors and pathways contribute to heart disease. Cells translate mechanical inputs into gene expression changes through integrated networks, including mechanosensitive non-coding RNAs. Human iPSC-derived cardiac cells, engineered heart tissues, and organoid models can complement animal heart models by enabling precise control of stiffness, strain, and shear while capturing human specific responses.

Key priorities are to map cardiac mechanical cues with higher spatial and temporal resolution and to link these maps to cell-type-specific responses. Regional mechanics vary across the cardiac wall and change during the cardiac cycle due to tissue viscoelasticity. Material properties also evolve with age and disease progression. For example, following MI, the fibrotic core, border zone, and remote myocardium exhibit distinct elastic moduli, reflecting spatial heterogeneity in tissue remodeling.^336^ Importantly, the consequences of mechanotransduction for the epigenetic landscape, cellular state plasticity, and long-term functional adaptation of cardiac cells remain incompletely understood. Even when exposed to similar mechanical stimuli, such as stretch, different cardiac cell types exhibit divergent responses. For instance, hypertrophic growth in cardiomyocytes vs myofibroblast transdifferentiation in fibroblasts. Likewise, inhibition of mechanosensors or mechanotransductive processes may produce cell-type-specific outcomes, including differential sensitivity to drug dosage and distinct fibrotic responses across cardiac cell types following Piezo1 inhibition.^271,286–288^ Moreover, mechanotransductive signaling networks are highly interconnected. For example, traction forces transmitted through integrins can activate latent TGF-β, while force transmission through the LINC complex can alter nuclear architecture and regulate YAP nuclear translocation and transcriptional activity. These layers of mechanical integration and signaling crosstalk must be carefully considered when targeting cardiac mechanotransduction for therapeutic intervention. Integrated approaches that combine advanced imaging, computational modeling, traction and calcium measurements, spatial transcriptomics, and single-cell multiomics will be instrumental in addressing these knowledge gaps and refining future strategies.

## Data Availability

Data sharing is not applicable to this article as no new data were created or analyzed in this study.
